# A review on hypersensitivity reactions to fungal aeroallergens in patients with allergic disorders in Iran

**DOI:** 10.18502/cmm.5.1.537

**Published:** 2019-03

**Authors:** Zeinab Nazari, Javad Ghaffari, Negar Ghaffari, Fatemeh Ahangarkani

**Affiliations:** 1 Department of Gynecology, Mazandaran University of Medical Sciences, Sari, Iran; 2 Pediatric Infecious Diseases Research Center, Mazandaran University of Medical Sciences, Sari, Iran; 3 Mazandaran University of Medical Sciences, Sari, Iran; 4 Invasive Fungi Research Center, Student Research Committee, Mazandaran University of Medical Sciences, Sari, Iran

**Keywords:** Allergic rhinitis, Asthma, Atopic dermatitis, Fungus, Mold, Urticaria

## Abstract

Fungal agents account for the clinical manifestation of allergic disorders. The aim of the present study was to review the prevalence of hypersensitivity reactions to fungal aeroallergens in patients with allergic disorders, including allergic rhinitis, asthma, urticaria, and eczema, in Iran. The initial literature search resulted in the identification of 50 records, 26 cases of which met the inclusion criteria. Regarding the methods adopted for the detection of fungal allergens, serum-specific IgE and skin prick tests were used in 6 and 20 studies, respectively. *Aspergillus fumigatus* and *Alternaria alternata* sensitization was the most common allergic sensitization among the patients with allergic disorders. According to the reviewed studies, despite the humid climate of the north of Iran, fungal sensitization has a prevalence range of 5-70% in this region. In other regions, such as central and southern Iran, which have a dry and warm climate, fungal sensitization reportedly has a prevalence range of 5-65%. The prevalence of fungal sensitizations varies in different allergic disorders due to the factors related to geographic and genetic issues, gender, sample size, test operator, and assessment method.

## Introduction

Fungal spores are broadly distributed in nature; accordingly, fungal exposure is a daily fact of human existence [1]. Fungal allergy is a common health problem around the world. Although in most of the studies, fungal allergens are introduced as the top causes of allergic disorders, they are among the most common risk factors for the exacerbation of allergic disorders [2]. It is estimated that 12-42%, up to 66%, and 3-10% of atopic patients, patients with severe asthma, and the general population may be sensitized to fungi, respectively [3]. Aspergillus, Alternaria, Cladosporium, Penicillium, and *Candida* species are the most common fungal allergens accounting for the development of allergic disorders. 

There are various reports regarding the distribution of fungal agents as allergens [4]. For example, although this fact is established that *Alternaria* species have a role in the pathogenicity of allergic disorders (e.g., allergic rhinitis), *Candida* and *Aspergillus* species have been identified as the most common sensitizing fungal allergens leading to allergic disorders [2, 5]. Allergic disorders, such as asthma, allergic rhinitis, atopic dermatitis, and urticaria, are common diseases around the world. 

The prevalence and incidence of aeroallergen sensitization vary across different countries and populations. In Iran, the prevalence rates of asthma are reported to be 2.7% (95% CI: 1.9-3.6) and 3.5% (95% CI: 2.6-4.6) in the children aged 6-7 and 13-14 years, respectively. Furthermore, in a study, the prevalence rates of ‘wheezing in the past 12 months’ were obtained at 7.6% (95% CI: 5.6-9.8) and 10.7% (95% CI: 8.9-12.7) in the children within the age range of 6-7 and 13-14 years, respectively [6]. 

Fungal allergenic sensitization is associated with the increased severity of asthma and hospital admission owing to the exacerbation of pulmonary disorders. The pooled prevalence rates of atopic dermatitis in the children aged 6 -7 and 13-14 years were reported as 5.98% and 6.52%, respectively [7]. However, in another study, the pooled prevalence rates of allergic rhinitis in children within the age of 6-7 and 13-14 years were 11.9% and 21.2%, respectively [8]. Nonetheless, the role of fungal exposure of outdoor samples in the development of childhood allergic rhinitis is poorly understood. It has been suggested that sensitization to *Alternaria*, *Aspergillus*, and *Penicillium* allergens is associated with allergic rhinitis and asthma exacerbation in pediatric patients [9, 10] 

Iran has different geoclimatic conditions; accordingly, the prevalence of aeroallergens, such as fungal agents, varies across different regions of this country. Allergic disorders are diagnosed based on patient’s history and physical examination, along with in vivo (i.e., skin prick test) and in vitro (i.e., radioallergosorbent test or immunoassay capture test [ImmunoCAP)] tests used as confirmatory assays [6-8, 11, 12]. Skin prick test has a high negative predictive value, while ImmunoCAP as a serum test for the detection of specific antibodies is more sensitive than the former [13]. According to the literature, in Iran, fungal allergens are mostly diagnosed by skin prick test. 

Allergen avoidance is the most effective way to manage allergic patients; nonetheless, it is usually difficult. Allergen-specific immunotherapy (ASIT) is an important therapeutic approach, which is administered for the patients that cannot be managed by conventional therapy [12]. However, there are insufficient documented data regarding the use of ASIT for the management of sensitization to fungal allergens. With this background in mind, the present review was conducted to investigate the evidence on the prevalence of hypersensitivity reactions to fungal aeroallergens in patients with allergic rhinitis, asthma, urticaria, and eczema in Iran. 

## Materials and Methods

For the purpose of the study, a comprehensive search was conducted in several databases, including PubMed, EMBASE, Google, SID, Magiran, and Irandoc, using the following keywords: “Skin prick test”, “Serum fungal-specific IgE”, “Fungal allergen”, “Aeroallergens”, “Asthma”, “Allergic rhinitis”, “Urticaria”, “Eczema”, and “Iran”. There was no time limit for searching the articles. The inclusion criteria were: 1) investigation of fungi in allergic diseases, 2) availability of the full-text version of the article, and 3) publication in English or Persian. On the other hand, the articles investigating issues other than allergic diseases or those with unclear methods were excluded from the study. 

## Results

The initial search process resulted in the identification of 50 records, 26 cases of which met the inclusion criteria. Regarding the methods adopted for the detection of fungal allergens, serum-specific IgE and skin prick tests were used in 6 and 20 studies, respectively. Tables 1 and 2 summarize the data related to the reviewed articles. Based on the results of the retrieved studies, the prevalence of fungal sensitization was obtained at 5-70% by skin prick test. The reasons accounting for this variability are due to the difference in sample size, type of test, type of allergen, and test operator. 

The prevalence of fungal sensitization is similar in the different areas of Iran. For example, in the north of Iran, fungal sensitization has a prevalence rate of 5-70% despite the warmness and humidity of this region. In the same vein, in the other areas, such as central and southern Iran, which have a dry and warm climate, the prevalence rate of fungal sensitization is estimated at 5-65%. It seems that geographic and climatic factors play a less significant role in sensitivity to fungal allergens. In a study performed in Mazandaran, Iran, the overall prevalence of sensitivity to fungal allergens, except for the *Candida* species (which was positive in 28% of the patients), among allergic patients was 0-10% [14]. In Esfahan, Iran, 37% of all patients tested positive for fungal allergens. 

**Table 1:** Characteristics of the included studies detecting fungal allergen by serum-specific IgE

**Number**	**First author [reference ]**	**Year**	**Serum-specific IgE target**	**Patients**	**Positive for fungal allergen (%)**	* **Location**	**Age**
1	Ghaderi [15]	2018	*Penicillium notatum* *Cladosporium herbarum* *Aspergillus fumigatus* *Alternaria alternata*	Atopic dermatitis	*Penicillium notatum *(93.5) *Cladosporium herbarum *(87.1), *Aspergillus fumigatus *(100) *Alternaria alternate *(96.8)	Birjand	15-82 years
2	Shokohi Shormasti [16]	2017	Mold	Allergic rhinitis, asthma, atopic dermatitis, and urticaria	(23.8 )	Tehran	10-60 years
3	Bonyadi [17]	2017	*Alternaria alternata* *Cladosporium herbarum*	Atopic dermatitis	*Alternaria alternate *(8) *Cladosporium herbarum* (6)	Tabriz	30.2±14.7 years
4	Khosravi [18]	2012	*Fusarium solani*	Asthma	(100)	Tehran	20-60 years
5	Khosravi [19]	2012	*Trichophyton mentagrophytes, Candida albicans, Aspergillus fumigatus*	Atopic cases	*Trichophyton mentagrophytes *(65.9), *Candida albicans *(17.1), *Aspergillus fumigatus *(4.9)	Tehran	15-80 years
6	Hedayati [20]	2009	*Alternaria alternata*	Atopic dermatitis and asthma	Atopic dermatitis (32) Asthma (38)	Sari	4 months to 60 years

* All cities are located in Iran.

**Table 2:** Characteristics of the included studies investigating fungal allergens in different allergic patients by skin prick test

**Number**	**Author/ [reference ]**	**Year**	**Patients**	**Positive for target fungal allergens (%)**	**Age**	* **Location**	**Most common allergen (%)**
1	Ahmadi Afshar [21]	2018	Allergic disorders	Molds (20.2)	23.9±0.6 years	Zanjan	Mites (23.9)
2	Yaghobi Oskoei [22]	2017	Allergic rhinitis	*Alternaria* spp. (5.72), *Aspergillus* spp. (3.14), *Cladosporium* spp. (0.2), and *Penicillium* spp. (0.4)	1-86 years	Mashhad	Russian thistle (54.6)
			Asthma	*Alternaria* spp. (8.58),* Aspergillus* spp. (5.52), *Penicillium* spp. (0.61)			Russian thistle (63.19)
			Urticaria	*Alternaria* spp. (2.6),* Aspergillus* spp. (0.86), *Penicillium* spp. (0.86)			Russian thistle (33.91)
			Atopic dermatitis	*Alternaria* spp. (5)			Russian thistle (42.5)
3	Pazoki [23]	2015	Allergic rhinitis	*Alternaria* spp. (31), *Aspergillus* spp. (18), *Cladosporium* spp. (3)	1-72 years	Tehran	Pollen (55)
4	Farokhi [24]	2015	Allergic rhinitis	*Alternaria* spp. (14-86)		Boushehr	Mites (88.5)
			Asthma	*Alternaria* spp. ( 14-52)			Mites (91)
5	Moghtaderi [25]	2015	Asthma	Molds (25 )	2-74 years	Shiraz	Pollen (47)
			Atopic dermatitis	Molds (11)			Pollen (58)
			Allergic rhinitis	Molds (14.7)			Pollen (66)
6	Shakornia [26]	2014	Allergic rhinitis, asthma, urticaria	*Alternaria* spp. (18.9), mold mix (9)	4-66 years	Ahwaz	Weeds (58)
7	Hosseini [27]	2014	Allergic rhinitis	*Alternaria* spp. (28)	4 months to18 years	Tehran	Tree mix (26)
			Asthma	*Alternaria* spp. (23)			
8	Shakornia [28]	2013	Allergic rhinitis and urticaria	*Cephalosporium acremontum* (11.5), mold mix (9.8), *Penicillium* spp. (9.5), *Alternaria* spp. (8.1), *A. fumigatus *(5.1)	4-70 years	Ahwaz	-
9	Mahram [29]	2013	Allergic rhinitis, asthma, atopic dermatitis	*Alternaria* spp. (27), *Penicillium* spp.(15.5)	24.6±1.26 years	Ghazvin	Weeds (59)
10	Ghaffari [14]	2012	Urticaria	*Aspergillus* spp. (1.3), *Alternaria* spp. (1.3)	7-50 years	Sari	Mites (36)
11	Ghaffari [30]	2011	Allergic rhinitis, asthma, urticaria	*Candida* spp. (28), *Penicillium* spp. (10), *Alternaria* spp. (4)		Sari	Mites (65)
12	Mokhtari- Amirmajdi [31]	2011	Allergic rhinitis	*Alternaria* spp. (45.5)	5-66 years	Mashhad	-
13	Nabavi [32]	2010	Asthma	*Aspergillus* spp. (7.2), *Penicillium* spp.(7.2), *Mucor* spp. (7.2), *Alternaria* spp. (5.14), *Cladosporium* spp. (2.13)	<18 years	Semnan	-
14	Ghaffari [33]	2010	Allergic rhinitis, asthma	*Alternaria* spp. (3.6), *Aspergillus* spp. (2.4)	5-50 years	Sari	Mites (50)
15	Moradi [34]	2010	Allergic rhinitis	*Alternaria *spp. (60), *Penicillium* spp. (54)	1-85 years	Boushehr	Mites (70)
			Asthma	*Penicillium* spp. (61), *Aspergillus* spp. (60)			Mites (72)
			Eczema	*Aspergillus* spp. (55), *Alternaria* spp. (54)			Mites (63)
			Acute urticaria	*Aspergillus* spp. (50)			Mites (67)
			Chronic urticaria	*Aspergillus* spp. (57.5)			Mites (71)
			Asthma	*Alternaria* spp. (23)			
16	Nabavi [35]	2009	Allergic rhinitis	*Aspergillus* spp. (12), *Cladosporium* spp. (11), *Alternaria* spp. (10.7), *Penicillium* spp. (8)	2-60 years	Semnan	-
17	Feridoni [36]	2009	Allergic rhinitis	*Aspergillus* spp. (8.6), *Alternaria* spp. (8.4), mold mix (6.3)	-	Mashhad	Weeds (77)
18	Khazaei [37]	2003	Allergic rhinitis, asthma, and urticaria	*Aspergillus* spp. (65),* Alternaria* spp. (57), *Cladosporium* spp. (47), *Penicillium* spp. (39)	2-79 years	Zahedan	Mites (90)
**Table 2. **Continued							
19	Kashef [38]	2003	Allergic rhinitis	Mixed fungus (9.8), *Alternaria* spp. (3.7), *Aspergillus* spp. (2.2), *Candida* spp. (0.7)	1-61 years	Shiraz	Pollens (92.4 )
20	Akbari Hedayati [39]	2000	Asthma	Molds (11)	-	Esfahan	Mites (38)

* All cities are located in Iran.

## Discussion

The findings of the reviewed studies were suggestive of the higher frequency of positive reactions to mold in females and adults as compared to those in males and other age groups in Iran, respectively. The results of some studies indicated no difference between males and females in terms of fungal sensitization [24, 28, 36]. Nonetheless, in other studies, mold sensitization rate was significantly different between males and females with asthma, atopic dermatitis, and chronic urticaria but not between those with acute urticaria [16, 34]. Shakurnia and Kashef et al. reported that mold sensitization was more common in males than in females [26, 38]. 

The results of some studies indicated the reduction of mold sensitization prevalence with aging [16, 26, 42], whereas a number of studies suggested the higher prevalence of fungal sensitization with the enhancement of age [25, 27, 38, 40]. Based on the evidence, mold sensitization was more common in individuals under the age of 20 years [16] and subjects within 15-35 years of age [26, 28]. 

Mahboubi et al. showed that fungal sensitization was more common in the summer; nonetheless, Shokohi Shormasti showed that fungal allergen sensitization was more common in the fall and winter [16, 22]. In addition, Hosseini et al. reported the higher prevalence of fungal allergen sensitization in the spring and summer [27]. Given the limited number of studies on the relationship between seasons and fungal sensitization, no conclusion can be made regarding the season inducing a higher predisposition to fungal sensitization. In dry and cold weather, mold sensitization was relatively more common in Zanjan (20%) and Esfahan (37%), respectively [21, 39]. As the evidence indicated, fungal sensitization was more common (55.6% and 82%) in the south of Iran (i.e., Bushehr) [24, 34] due to the high humidity of this region; however, it was less common in Shiraz, Iran (10.9%) [25]. 

In asthmatic patients, hypersensitivity reactions to molds had a prevalence rate of 11%, and mold sensitization was more common in the autumn and winter than in the other seasons [39]. In a study performed in Shiraz, the prevalence of mold hypersensitivity reactions was reported as 8.3% in allergic rhinitis patients. In this regard, mixed fungi (9.8%) were identified to be more common, followed by *Alternaria* species (3.7%), *Aspergillus* species (2.2%), and *Candida* species (0.7%). In a couple of reviewed articles, swamp coolers and new air conditioners were introduced as the main causes of fungal sensitization in dry and warm weather [38, 25].

Amirmajdi et al. determined the prevalence of hypersensitivity reaction to *Alternaria* species as about 45.5% among allergic rhinitis patients by skin prick test. They also reported a significant relationship between *Alternaria* species and allergic rhinitis. In addition, they observed an association between *Alternaria* species sensitization and the isolation of *Alternaria* species from nasal discharge. In the mentioned study, direct examination and culture of *Alternaria* species were positive in 32.8% and 43.1% of the cases, respectively [31]. In a study conducted by Hedayati, the positive results were more frequent in the direct microscopic examination of mold allergen (70%) than those obtained from culture (40%) [40]. 

In dry climates (all regions in Iran, except for the northern part), pollen aeroallergens are more common than mold allergens. Mites were reported as the most common allergens in the different regions of Iran, except for Zahedan despite its dry and warm climate [37]. Mites are more common in a moderate temperature with high humidity, such as that of the north of Iran [4]. In all cases of allergic disorders, sensitization to *Aspergillus fumigatus* and *Alternaria alternata* was more common [15-39]. Furthermore, *Aspergillus fumigatus* and *Alternaria alternata* sensitization was more common in allergic rhinitis patients. Additionally, sensitization to *Aspergillus fumigatus* and *Penicillium* species was more common in patients with asthma disorder, while those with urticaria and atopic dermatitis showed more sensitization to *Aspergillus fumigatus* [15-39].

However, out of all fungal allergens, *Aspergillus fumigatus* sensitization was the most common allergic sensitization in allergic disorders. Based on the reviewed articles, fungal allergens that tested positive in allergic disorders included *Aspergillus fumigatus*, *Alternaria alternata*, *Penicillium notatum*, *Candida* species, *Cladosporium* species, and *Rhizopus* species. Exposure to *Alternaria* species is an important risk factor for the prevalence of asthma and can lead to severe and potentially fatal asthma [41]. 

*Alternaria* species sensitization and presentation of *Alternaria* species in the upper respiratory tract might induce allergic rhinitis. Nonetheless, there was no significant relationship between *Alternaria* species sensitization and severity of allergic rhinitis [31]. Severity of *Alternaria* species sensitization had a direct association with more severe asthma [34]. Nonetheless, no such relationship was detected in another study [24]. Mold sensitization was more common in seasonal allergic rhinitis than in perennial allergic rhinitis; however, it was not statistically significant. 

In addition, mold sensitization was not different between intermittent and persistent or between mild and moderate to persistent allergic rhinitis [24, 42]. 

Studies showed that mold sensitization was more common in asthmatic patients than in patients with allergic rhinitis, atopic dermatitis, and asthma plus allergic rhinitis [25, 27]. Nevertheless, in a study carried out by Ghaffari, mold sensitization was more common in asthmatic patients than in patients with allergic rhinitis or urticaria [30]. 

The results of a couple of studies were indicative of a significant relationship between mold sensitization and severity of allergic rhinitis [24, 34]. A study suggested an increased rate of mold sensitization from 8.3% to 14.7% during a decade among allergic rhinitis patients [25]. Ahmadiafshar et al. showed that dyspnea, nasal discomforts, and eye manifestations were significantly more common in patients who were positive for mold sensitization [21]. 

The evidence revealed no significant difference between the two genders and different age groups with allergic disorders in terms of mold sensitization [34, 36, 38]. Mold sensitizations are more common in tropical countries, such as Malaysia and Singapore [43, 44]. In Malaysia, *Fusarium* species (23.5%), *Aspergillus flavus* (21.2%), *Dreselera orysae* (18.8%), *Alternaria* species (17.6%), *Curvularia eragrostidis* (17.6%), *Penicillium* species (16.5%), *Pestolotriopsis gtuepini* (16.5%), *Rhizopus arrhizus* (16.5%), *Aspergillus* *niger* (15.3%), *Penicillium choy* (12.9%), *Aspergillus fumigatus* (11.8%), and *Cladosporium* species (4.7%) were detected by skin prick test reactivity [43]. 

## Conclusion

The prevalence of fungal sensitizations is different in allergic disorders due to the factors related to geographic and genetic issues, gender, sample size, participants’ age, climatic region, test operator, and assessment method. All fungal allergens are relatively common in the different regions of Iran. Sensitization to a few fungi, such as *Alternaria* species, is correlated with the severity of asthma. Consequently, fungal allergen should be considered in patients with allergic disorders. 
